# Machine learning for identifying tumor stemness genes and developing prognostic model in gastric cancer

**DOI:** 10.18632/aging.205715

**Published:** 2024-04-12

**Authors:** Guo-Xing Li, Yun-Peng Chen, You-Yang Hu, Wen-Jing Zhao, Yun-Yan Lu, Fu-Jian Wan, Zhi-Jun Wu, Xiang-Qian Wang, Qi-Ying Yu

**Affiliations:** 1 Department of Oncology and Central Laboratory, Tumor Hospital Affiliated to Nantong University, Nantong, Jiangsu 226361, P.R. China; 2 Department of Oncology, The Affiliated Hospital of Nantong University, Nantong, Jiangsu 226361, P.R. China; 3 Institute of Biology and Medicine, College of Life and Health Sciences, Wuhan University of Science and Technology, Wuhan, Hubei 430081, P.R. China; 4 Department of Oncology, Nantong Hospital of Traditional Chinese Medicine, Nantong, Jiangsu 226361, P.R. China

**Keywords:** gastric cancer, cancer stemness, machine learning, ssGSEA

## Abstract

Gastric cancer presents a formidable challenge, marked by its debilitating nature and often dire prognosis. Emerging evidence underscores the pivotal role of tumor stem cells in exacerbating treatment resistance and fueling disease recurrence in gastric cancer. Thus, the identification of genes contributing to tumor stemness assumes paramount importance. Employing a comprehensive approach encompassing ssGSEA, WGCNA, and various machine learning algorithms, this study endeavors to delineate tumor stemness key genes (TSKGs). Subsequently, these genes were harnessed to construct a prognostic model, termed the Tumor Stemness Risk Genes Prognostic Model (TSRGPM). Through PCA, Cox regression analysis and ROC curve analysis, the efficacy of Tumor Stemness Risk Scores (TSRS) in stratifying patient risk profiles was underscored, affirming its ability as an independent prognostic indicator. Notably, the TSRS exhibited a significant correlation with lymph node metastasis in gastric cancer. Furthermore, leveraging algorithms such as CIBERSORT to dissect immune infiltration patterns revealed a notable association between TSRS and monocytes and other cell. Subsequent scrutiny of tumor stemness risk genes (TSRGs) culminated in the identification of CDC25A for detailed investigation. Bioinformatics analyses unveil CDC25A’s implication in driving the malignant phenotype of tumors, with a discernible impact on cell proliferation and DNA replication in gastric cancer. Noteworthy validation through *in vitro* experiments corroborated the bioinformatics findings, elucidating the pivotal role of CDC25A expression in modulating tumor stemness in gastric cancer. In summation, the established and validated TSRGPM holds promise in prognostication and delineation of potential therapeutic targets, thus heralding a pivotal stride towards personalized management of this malignancy.

## INTRODUCTION

Gastric cancer stands out for its high morbidity and mortality rates among malignancies [1, 2]. Despite advancements in stomach cancer therapies in recent decades, current treatment modalities often fall short of achieving optimal outcomes, presenting significant risks to patients’ health and longevity [3–6]. Hence, a comprehensive understanding of the molecular mechanisms underpinning gastric cancer’s pathogenesis and progression, coupled with the exploration of innovative treatment strategies and reliable prognostic indicators, emerges as a pressing imperative.

Gastric cancer exhibits significant heterogeneity, and mounting evidence underscores the pivotal role of cancer stem cells (CSCs) in driving this diversity [7]. This heterogeneity presents formidable challenges for current treatment modalities. Within tumors, CSCs represent a distinct subpopulation, albeit constituting a minority. Possessing stem cell-like characteristics including self-renewal, replication, and differentiation capabilities [8, 9], CSCs perpetuate tumor progression through continual proliferation and differentiation. Remarkably, CSCs are intricately linked to key facets of gastric cancer pathogenesis, encompassing metastasis, drug resistance, and recurrence [10–12]. Consequently, precise elucidation of tumor stemness genes and their regulatory networks is imperative for accurate prognosis and targeted therapeutic interventions in gastric cancer management.

To preliminarily screen for tumour stemness genes in gastric cancer, two methods were employed. The first involved utilising the WGCNA algorithm and mRNAsi features for module distinction, while the second entailed adopting the ssGSEA method to score TCGA samples and obtain differential genes using the embryonic stem cell gene set as a criterion. The objective analysis of both methods yielded promising results. The genes acquired from both algorithms are intersected, and the resulting genes undergo various machine learning screenings to identify TSKGs. The machine learning outcomes, as screened by a confusion matrix and ROC, were utilised to develop a tumour stemness risk prognostic model. Cox’s regression analysis was carried out to verify the predictive value of the tumour stemness risk score (TSRS). We used ssGSEA and other algorithms, including CIBERSORT, to examine the correlation between the TSRS and tumour immune infiltrating cells. We analysed the expression level and tumour stemness index relationship and chose CDC25A as our research subject to investigate its expression, mutational landscape and functional enrichments in gastric cancer. We conducted assays on cell activity, cell cycle and immunofluorescence to confirm the function of CDC25A in gastric cancer, with specific focus on cell proliferation, cell cycle and tumour stemness. Our study had, in summary, established a reliable prognostic model in tumour stemness. The model presented here could strengthen the development of targeted therapy for tumour stem cells in gastric cancer. Additionally, it provides a theoretical basis for precise and personalized patient treatment.

## MATERIALS AND METHODS

### Cell culture

The SGC7901 gastric cancer cells utilized in this investigation were procured from the Chinese Academy of Sciences Cell Bank (Shanghai, China). These cells were maintained in RPMI-1640 medium supplemented with 10% fetal bovine serum (FBS) and 1% penicillin/streptomycin.

### Cellular immunofluorescence

Please refer to our previously published literature for specific experimental procedures [13]. The antibodies CD44 and Ki67 in the experiments were from ABclonal (USA).

### Flow cytometry for cell cycle detection

The quantification of various cell cycle phases was conducted using a cell cycle detection kit (HY-K1071, MCE, USA) in accordance with the manufacturer’s instructions. Following treatment, cell samples were subjected to analysis via flow cytometry using a BD C6 plus instrument.

### Immune infiltration analysis

Tumor purity was assessed using transcriptomic profiles of gastric cancer cohorts from TCGA with the ESTIMATE algorithm. ImmuneScore, StromalScore, and ESTIMATEScore were also compared between the low- and high-TSRS groups. ssGSEA was performed to analyze 28 immune cells using the GSVA package. We then validated these results with pan-cancer immune infiltration data and XCELL, CIBERSORT-ABS, EPIC, TIMER and etc., algorithms [14].

### Identification of TSKGs

In this analytical segment, two distinct algorithms were employed to identify tumor stemness genes within gastric cancer, followed by an intersection analysis of their results. Initially, the WGCNA algorithm, coupled with mRNAsi, was utilized to compute tumor stemness gene modules and subsequently extract genes of interest from these modules. Subsequently, the second algorithm leveraged the MSigDB database to download embryonic stem cell genomes, utilizing them as scoring criteria via the GSVAR software package to score gastric cancer samples sourced from TCGA. Following this, the limma package facilitated the classification of samples into high and low groups, enabling the identification of differentially expressed genes. Finally, the outcomes from both algorithmic approaches were subjected to intersection analysis using the R package venn.

### Machine learning

In this segment of the analysis, we applied five distinct machine learning algorithms to identify TSKGs. The utilized algorithms encompass Support Vector Machine (SVM), K-Nearest Neighbors (KNN), Decision Tree (DT), Logistic Regression (Logit), and Random Forest (RF). The top 30 genes, ranked by their importance, were visually represented through bar charts for each algorithm. Subsequently, the efficacy of these results was assessed through accuracy testing, employing methodologies such as confusion matrices and ROC curves. Noteworthy R language packages utilized in this process include caret, DALEX, ggplot2, random Forest, kernlab, and pROC.

### Analyses of mutated landscapes

Mutational landscapes of the CDC25A high and low expression groups were mapped by applying the website assistant for clinical bioinformatics (https://www.aclbi.com/static/index.html#/).

### Statistical analysis

The unpaired *T*-test was used to calculate the *p*-value, and *p* < 0.05 was considered statistically significant.

### Availability of supporting data

The data generated during this study are included in this article and its supplementary information files are available from the corresponding author on reasonable request.

## RESULTS

### ssGSEA and WGCNA were used to screen the tumor stemness genes of gastric cancer

**Figure 1:**
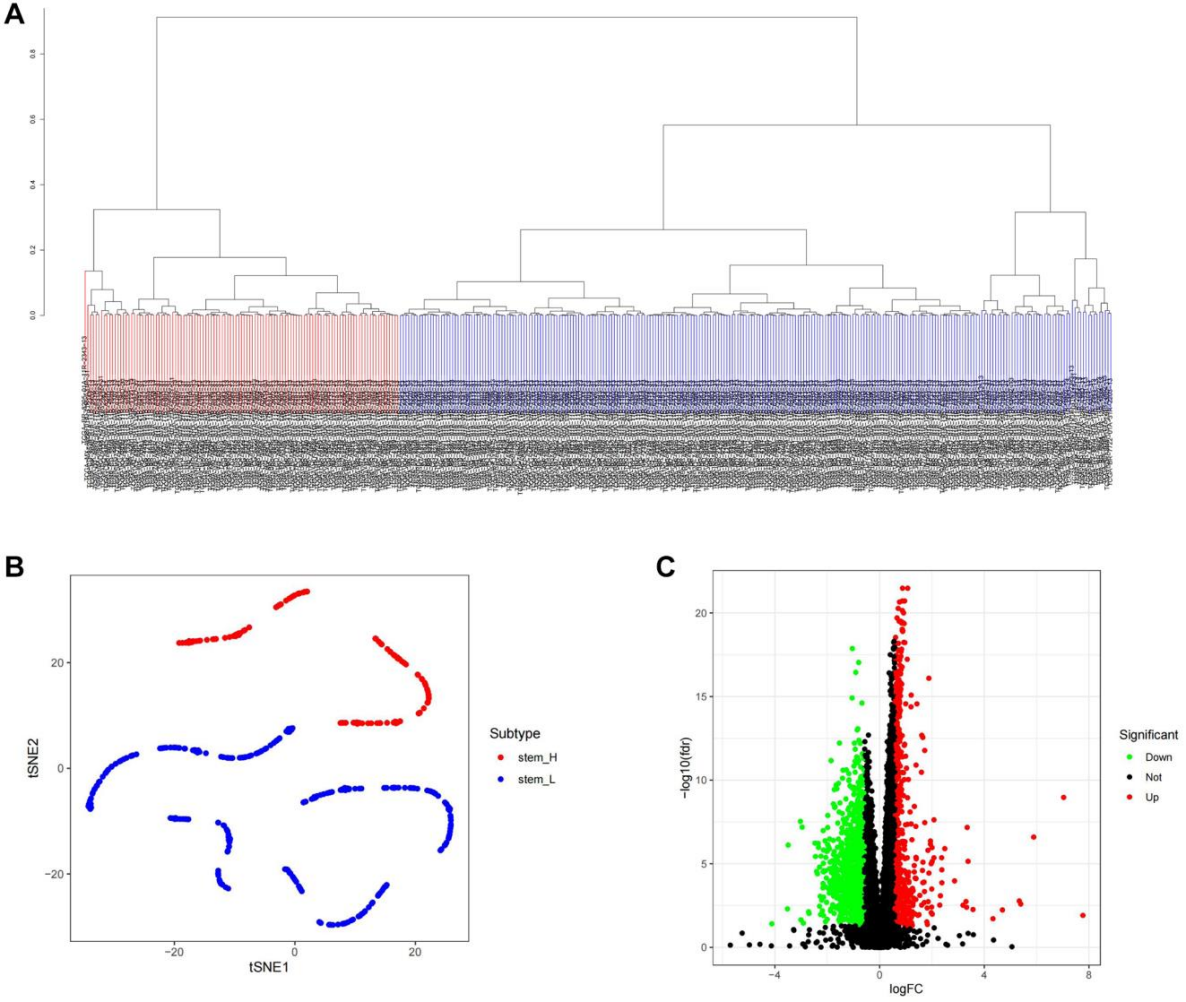
**ssGSEA algorithm was used to identify the differential genes of tumor stemness in TCGA samples.** (**A**) Distribution map of high and low score groups of TCGA samples. (**B**) tSNE was used to analyze the distribution between high and low stemness score groups. (**C**) Volcano plot of differentially expressed genes between high and low stemness score groups.

mRNAsi serves as a reference standard for assessing stemness in tumor samples [15]. Initially, we compared the distribution of values between cancer and paracancer tissues, as illustrated in Supplementary Figure 1A, and observed a significant elevation in mRNAsi levels within cancerous tissue. Subsequently, we conducted an analysis of gastric cancer samples from TCGA to identify DEGs, as depicted in Supplementary Figure 1B, 1C. WGCNA was then employed on the DEGs to delineate the module exhibiting the highest positive correlation with mRNAsi. During the WGCNA analysis, we set the threshold for excluding outlier samples at 12,000 (Supplementary Figure 2A), established the R2 value for achieving a scale-free network at 0.9, and set β to 3 (Supplementary Figure 2B). The height for merging similarity modules was set at 0.35 (Supplementary Figure 2C), and the resultant merged modules were visualized on a Cluster Dendrogram (Supplementary Figure 2D). Subsequently, we identified a positive correlation involving nine modules. This correlation was then depicted on a heatmap with respect to mRNAsi and EREG-mRNAsi (Supplementary Figure 2E). The blue module was selected for gene extraction, yielding 694 genes (Gene Significance = 0.2, Module Membership = 0.2). Tumor stem cells represent a distinct class with stem cell-like properties. To assess and score TCGA samples for stem cell characteristics, we utilized the embryonic stem cell dataset from the Gene Set Enrichment Analysis (GSEA) database and applied the ssGSEA algorithm. Following scoring of each sample, the sample population was stratified based on the median score, as depicted in Figure 1A. The t-Distributed Stochastic Neighbor Embedding (tSNE) algorithm was employed to visualize the classification results, revealing distinct clustering between high and low stemness score groups (Figure 1B). Additionally, we analyzed the differentially expressed genes within the clustered sample groups, as presented in Figure 1C.

### Machine learning was used to screen the key genes of tumor stemness

**Figure 2:**
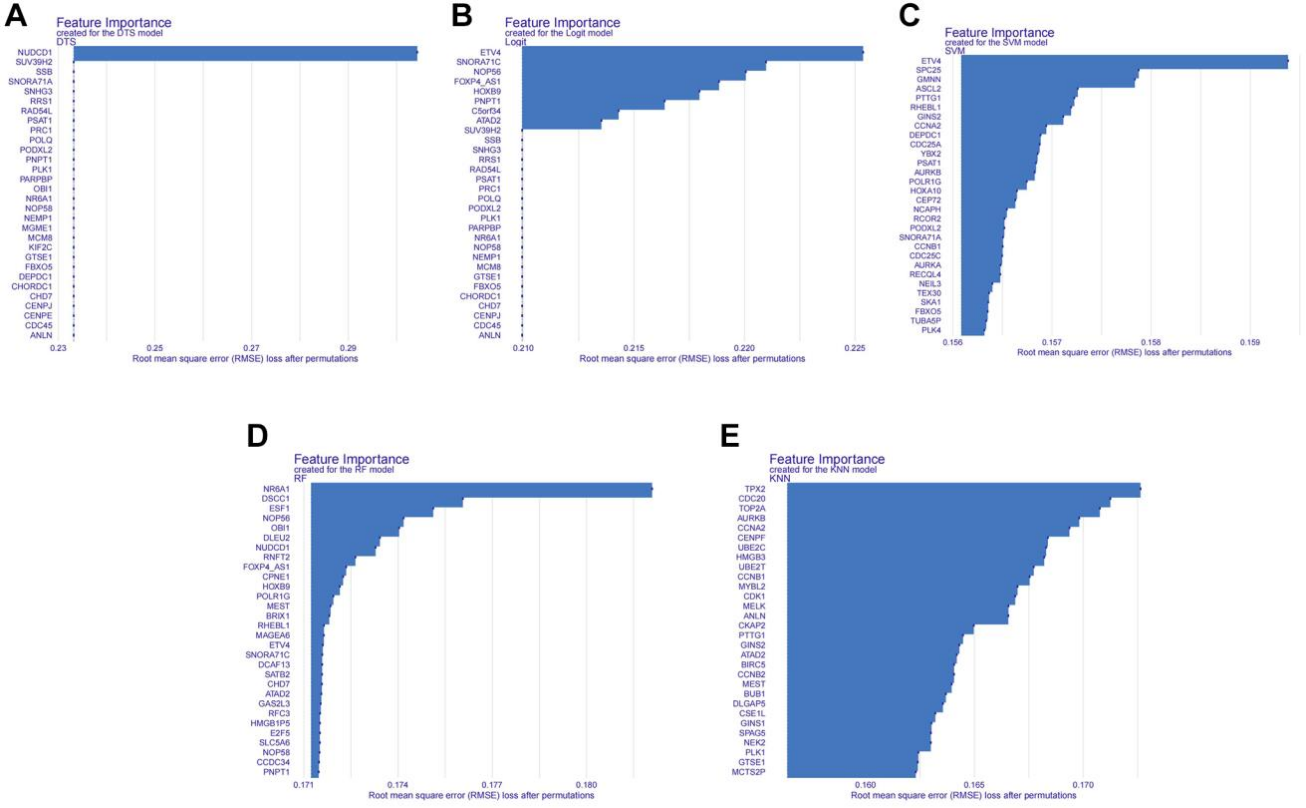
**Histogram of the importance of genes in different machine learning algorithms.** (**A**) Histogram of gene importance for the DTS machine learning algorithm. (**B**) Histogram of gene importance for the Logit machine learning algorithm. (**C**) Histogram of gene importance for the SVM machine learning algorithm. (**D**) Histogram of gene importance for the RF machine learning algorithm. (**E**) Histogram of gene importance for the KNN machine learning algorithm.

**Figure 3:**
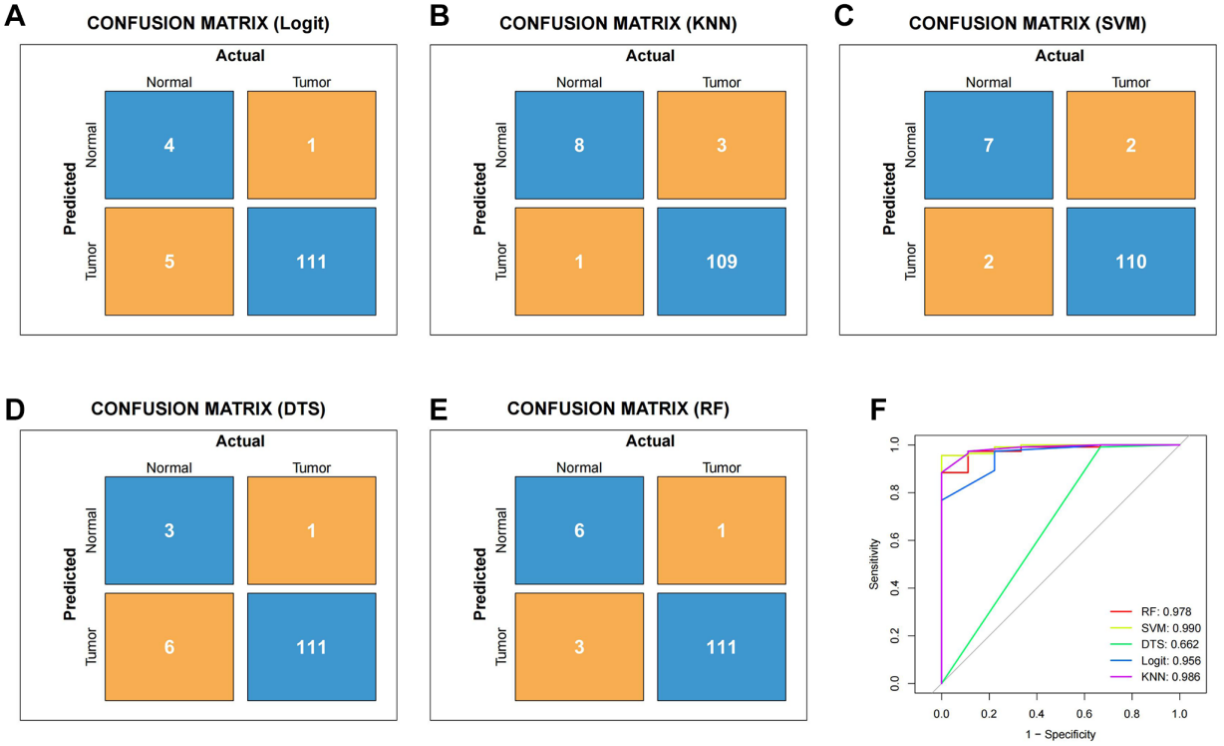
**To verify the reliability of different machine learning algorithms.** (**A**) Confusion matrix of Logit machine learning algorithm. (**B**) Confusion matrix of KNN machine learning algorithm. (**C**) Confusion matrix of SVM machine learning algorithm. (**D**) Confusion matrix of DTS machine learning algorithm. (**E**) Confusion matrix of RF machine learning algorithm. (**F**) The ROC curve of different machine learning.

We conducted a Venn analysis of the tumour stemness genes acquired via the algorithms WGCNA and ssGSEA, yielding a total of 182 genes (Supplementary Figure 3). Machine learning has extensive use in identifying significant genes. Therefore, we utilized diverse algorithms to identify key genes associated with tumour stemness. We categorised 182 genes into SVM, KNN, DTS, RF and Logit algorithms. The top 30 genes of importance were plotted in histograms for each algorithm (Figure 2A–2E). Subsequently, we evaluated the results from each algorithm to achieve better accuracy in identifying tumour stemness genes. As demonstrated in Figure 3A–3E, the confusion matrix was utilized to calculate the results. The SVM confusion matrix displayed values of 2 in the tumour diagonal module, indicating the SVM algorithm had the lowest error rate among the five machine learning algorithms evaluated. To ensure the reliability of the findings, we used the ROC curve to authenticate our results. The ROC analysis indicated that the SVM had an optimal AUC score of 0.990 (Figure 3F). Based on these results, we chose to investigate the role of tumour stem cells in gastric cancer using the SVM-calculated results for further in-depth study.

### Construction and evaluation of tumor stemness gene prognostic model

**Figure 4:**
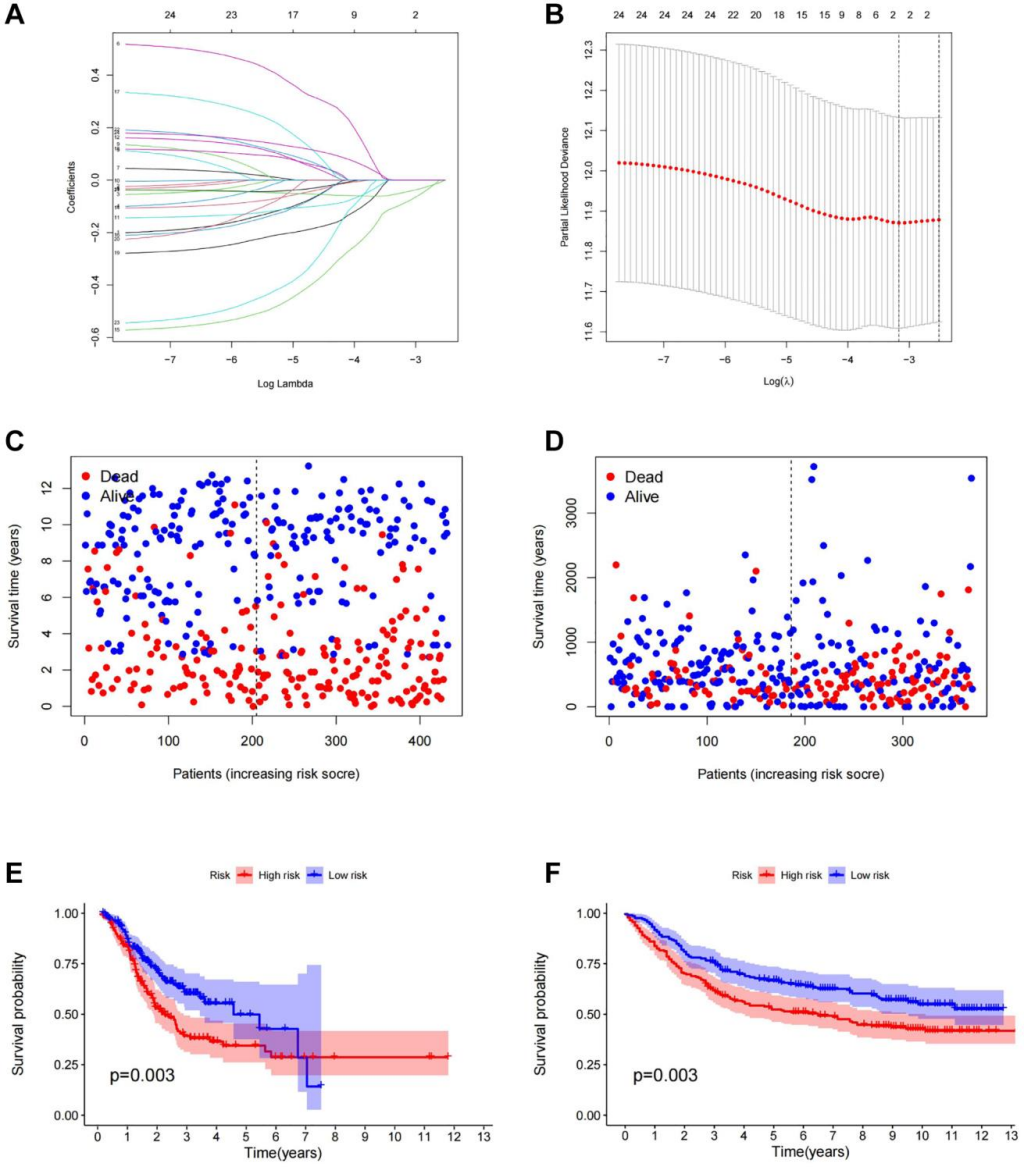
**Construction of prognostic model.** (**A**) LASSO coefficient profiles of the genes obtained from the SVM machine learning algorithm. (**B**) Partial likelihood deviance was plotted versus log (Lambda). The vertical dotted line indicates the lambda value with the minimum error and the largest lambda value. (**C**) The distribution of survival status in TCGA cohorts based on TSRS. (**D**) The distribution of survival status in GEO cohorts based on TSRS. (**E**) The patient samples from TCGA were divided into high and low TSRS groups and the OS of the groups were analyzed. (**F**) OS analysis of high and low TSRS groups from the GEO samples. Abbreviation: LASSO: least absolute shrinkage and selection operator.

Cancer stem cells represent the degree of malignancy of tumors and are associated with poor prognosis of patients. Therefore, it is of great significance to construct a prognostic model related to tumor stemness for accurate treatment of patients’ conditions. The outcomes obtained by SVM were classified into the LASSO algorithm (Figure 4A, 4B). We evaluated the model by using the GEO gastric cancer dataset GSE84437 as a test dataset to validate the results.

**Figure 5:**
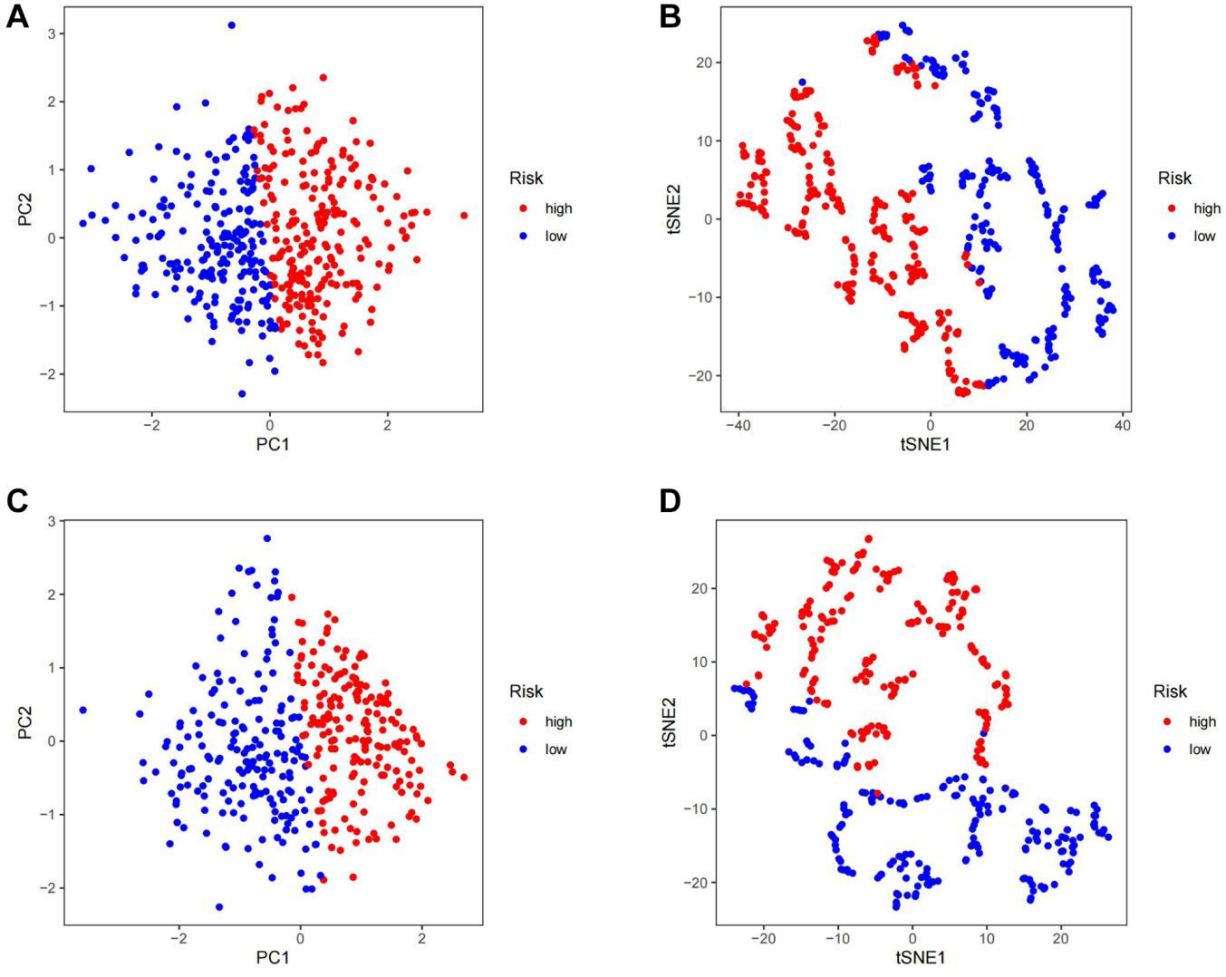
**Assess the ability of TSRS to differentiate patients in high and low TSRS group.** (**A**) PCA assessed the ability of TSRS to differentiate between high and low TSRS groups of patients in the TCGA cohorts. (**B**) tSNE assessed the ability of TSRS to differentiate between high and low TSRS groups of patients in the TCGA cohorts. (**C**) PCA assessed the ability of TSRS to differentiate between high and low TSRS groups of patients in the GEO cohorts. (**D**) tSNE assessed the ability of TSRS to differentiate between high and low TSRS groups of patients in the GEO cohorts.

**Figure 6:**
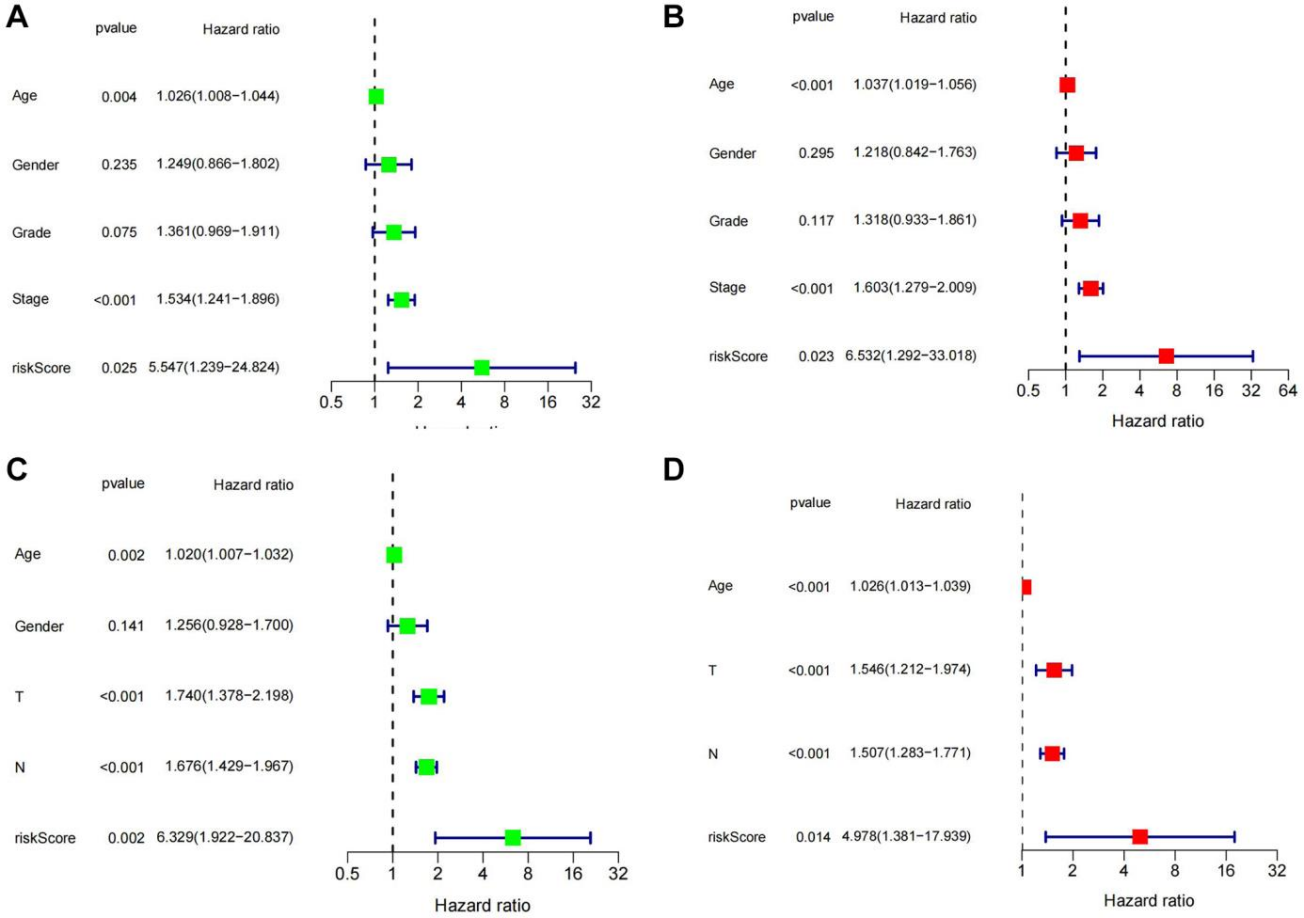
**Assessment of the independent prognostic role of TSRS.** (**A**, **B**) Univariate and Multivariate Cox analysis of TSRS and clinical characteristics in TCGA. (**C**, **D**) Univariate and Multivariate Cox analysis of TSRS and clinical characteristics in GEO.

Firstly, gastric cancer samples were classified into high and low TSRS groups based on their TSRS. The TSRS and survival status of the samples were then plotted as scatter plots to assess whether survival changed with increasing TSRS. Figure 4C, 4D indicated that the number of deaths increased in the high TSRS group, suggesting that patients in this group may have an unfavorable prognosis. To confirm this hypothesis, we conducted a Kaplan-Meier analysis on patients with high and low TSRS. The outcomes demonstrated that those with higher TSRS suffered significantly shorter survival (refer to Figure 4E, 4F). The tSNE and PCA findings in the testing and training sets indicated that TSRS could effectively differentiate the risk of the sample population (refer to Figure 5A–5D). Consequently, we suggested that TSRS could function as an independent prognostic factor. Next, following COX regression analysis, it was discovered that TSRS displayed a significant correlation with OS in both the training (Figure 6A, 6B) and testing sets (Figure 6C, 6D).

**Figure 7:**
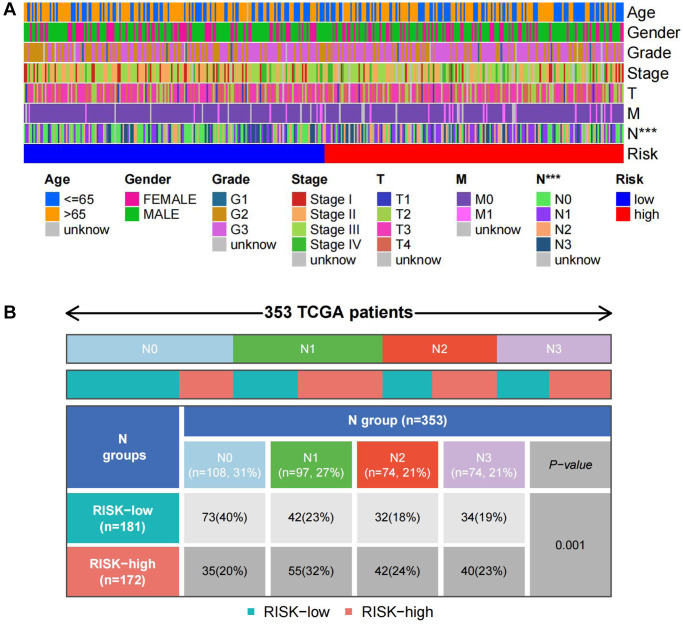
**Distribution of TSRS in different pathological parameters.** (**A**) Clinical heat map of clinicopathological parameters and TSRS. (**B**) Clinical heat map of N and TSRS.

**Figure 8:**
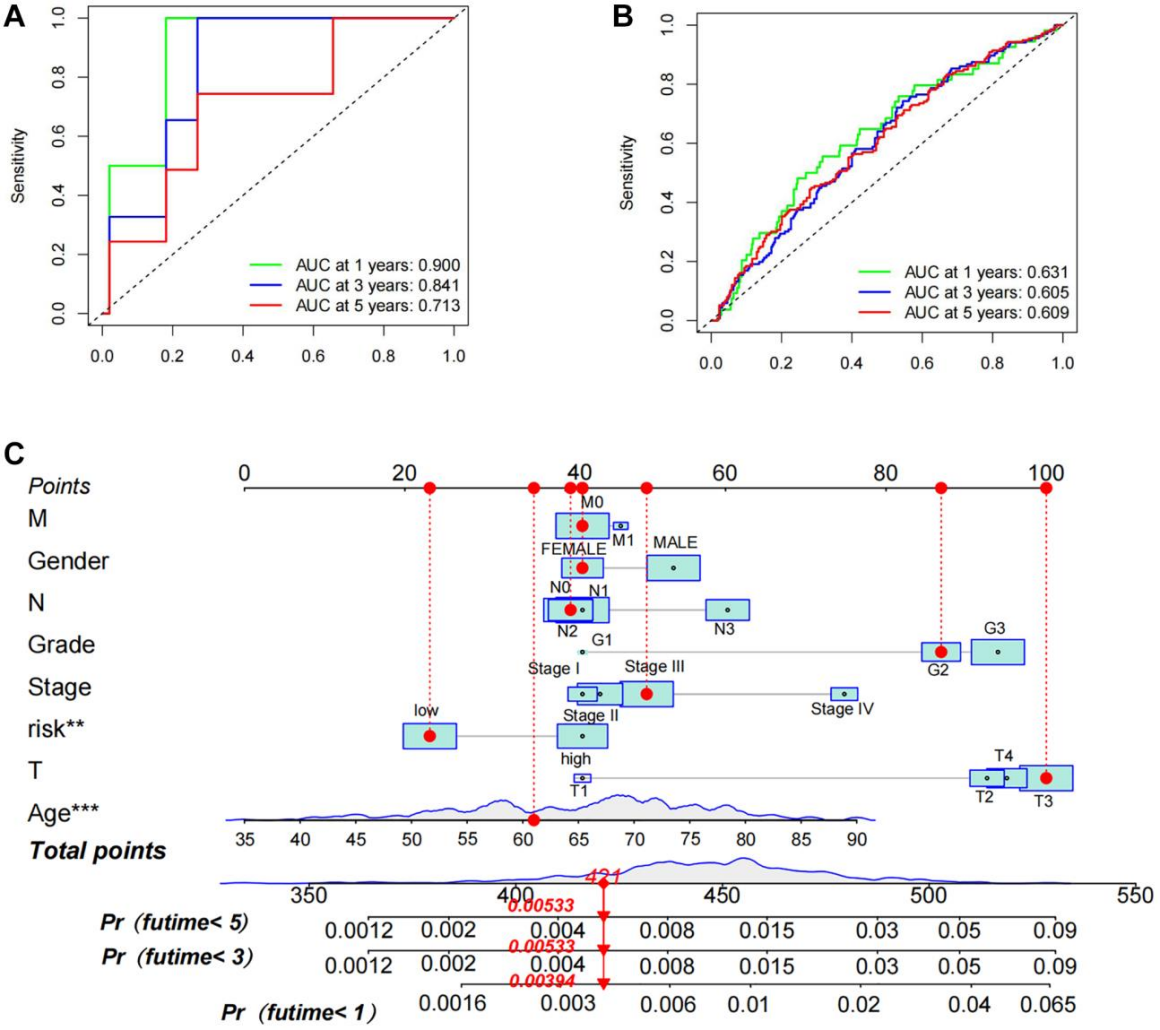
**Assessment of prognostic accuracy of TSRS as well as nomogram construction.** (**A**) ROC curve analysis in TCGA. (**B**) ROC curve analysis in GEO. (**C**) A nomogram constructed using TSRS and other parameters.

To explore the prognostic significance of TSRS in gastric cancer and its clinical implications, an analysis of TSRS distribution across various clinicopathological parameters was conducted. Our findings revealed notable disparities in the N pathological parameter concerning TSRS (Figure 7A). Subsequent assessment delineated the specific distribution of TSRS within the N pathological parameter (Figure 7B). Remarkably, the low TSRS cohort exhibited a predominant proportion of No patients (73 patients, 43%), while the high TSRS group displayed a higher incidence of N1 patients (55 patients, 32%). Furthermore, the prognostic efficacy of TSRS was evaluated across both the training (Figure 8A) and test sets (Figure 8B). Leveraging results from ROC analysis, a nomogram was constructed to harness the prognostic utility of TSRS, facilitating tailored treatment recommendations for patients (Figure 8C).

### Analysis of tumour immune cell infiltration in different TSRS subgroups

The tumor microenvironment (TME) plays a pivotal role not only in the initiation of early tumorigenesis and distant metastasis but also in its dynamic alterations as the tumor progresses [16, 17]. Within this complex milieu, cytokines secreted by immune cells associated with the tumor promote the survival and differentiation of tumor stem cells, among other processes. Conversely, tumor stem cells themselves secrete chemokines that recruit immune cells to the tumor site [18]. In our study, we conducted a comprehensive analysis of immune infiltrating cells in TSRS subgroups.

**Figure 9:**
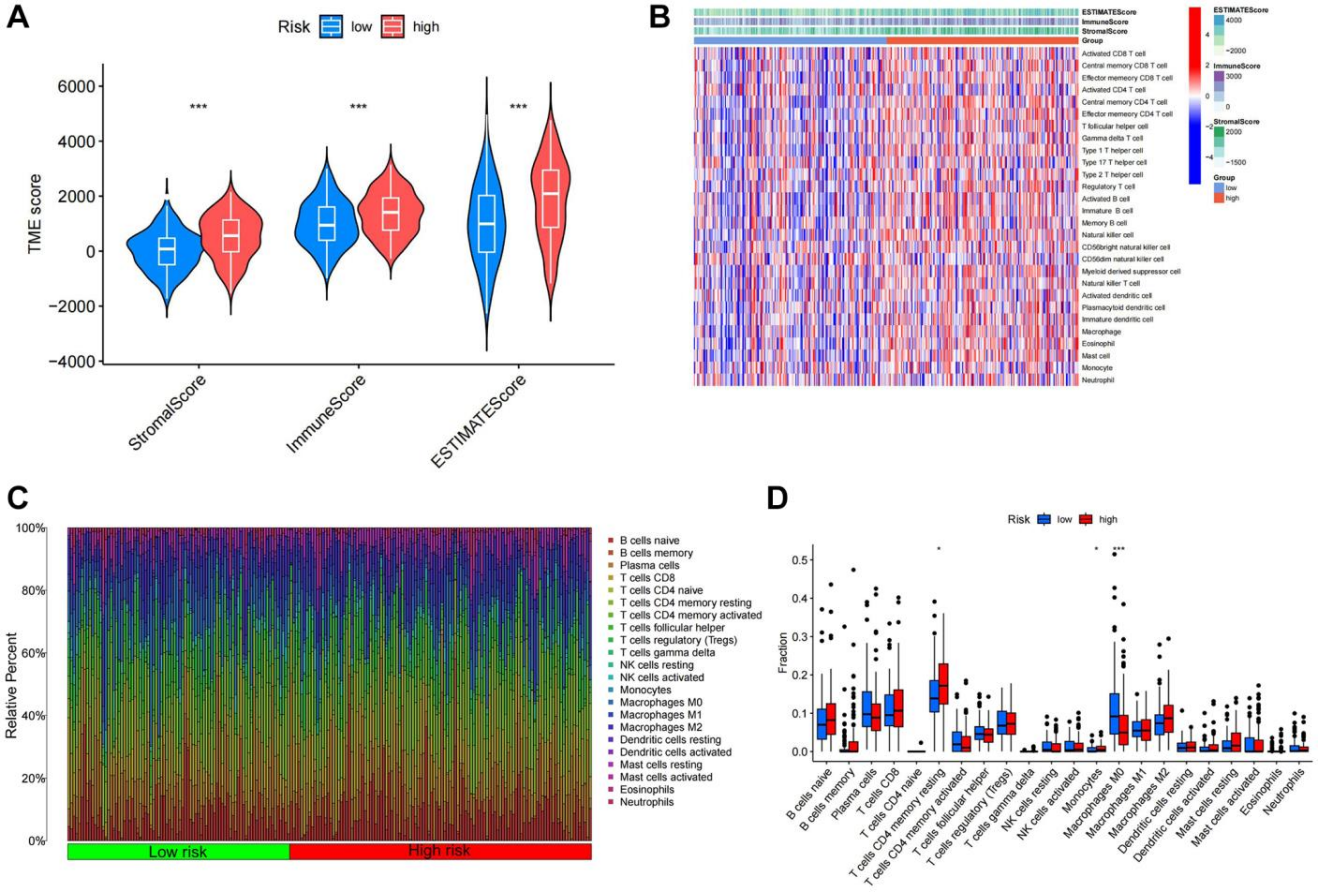
**Immune infiltration analysis of different TSRS groups.** (**A**) ESTIMATE analysis of high and low TSRS groups. (**B**) ssGSEA analysis of high and low TSRS group. (**C**) Histogram of different immune cell content in each sample. (**D**) Comparison of the content of different immune cells in high and low TSRS groups.

Employing the ESTIMATE algorithm, we first assessed tumor purity across different TSRS groups (Figure 9A). Subsequently, utilizing ssGSEA, we scrutinized and visualized the distribution of 28 immune infiltrating cells within both high and low TSRS groups through heat map. While no significant variations were observed in the distribution of these cells between the two groups, there was a discernible preference of immune-infiltrating cells within the high TSRS group (Figure 9B). These findings further reinforce the outcomes derived from the ESTIMATE ImmuneScore analysis.

We employed CIBERSORT to quantify immune-infiltrating cell content, uncovering significant associations within the Tumor Stemness-Related Signature (TSRS) cohorts. Specifically, T cell CD4+ memory resting and monocytes exhibited elevated levels in the high TSRS group, while macrophage M0 levels were heightened in the low TSRS group (Figure 9C, 9D).

To corroborate these findings, we utilized data from the pan-cancer database and various algorithms. XCELL analysis revealed significant correlations between monocytes and T cell CD4+ memory resting with TSRS, while macrophage M0 showed no such association (Supplementary Figure 4A). Monocytes consistently exhibited an association with TSRS across multiple algorithms including XCELL, QUANTISEQ, CIBERSORT-ABS, and MCPCOUNTER (Supplementary Figure 4A, 4C–4E).

Notably, macrophage M2 displayed a significant association with TSRS according to outcomes from the XCELL, QUANTISEQ, and CIBERSORT-ABS algorithms. Conversely, in TIMER and EPIC analyses, only macrophages and T-cell CD4+ were linked with TSRS, without identifying specific cellular subpopulations (Supplementary Figure 4B, 4F). These comprehensive analyses shed light on the intricate interplay between immune cell compositions and TSRS, offering valuable insights into tumor microenvironment dynamics.

Immune checkpoints play a critical role in regulating immune responses, ensuring autoimmune tolerance, and moderating the duration and intensity of immune reactions within tumor tissues. Consequently, we delved into the expression patterns of immune checkpoints across different subgroups of TSRS. As depicted in Supplementary Figure 5, all immune checkpoint genes exhibited heightened expression levels in the high TSRS group. These included genes associated with immune evasion, exhaustion, and suppression, alongside those activating immune cell cytotoxicity. These findings suggest robust activation of immune cells within the high TSRS subgroup, potentially predisposing them to immune exhaustion.

### Expression analysis of CDC25A in gastric cancer and its relationship with malignant phenotype

**Figure 10:**
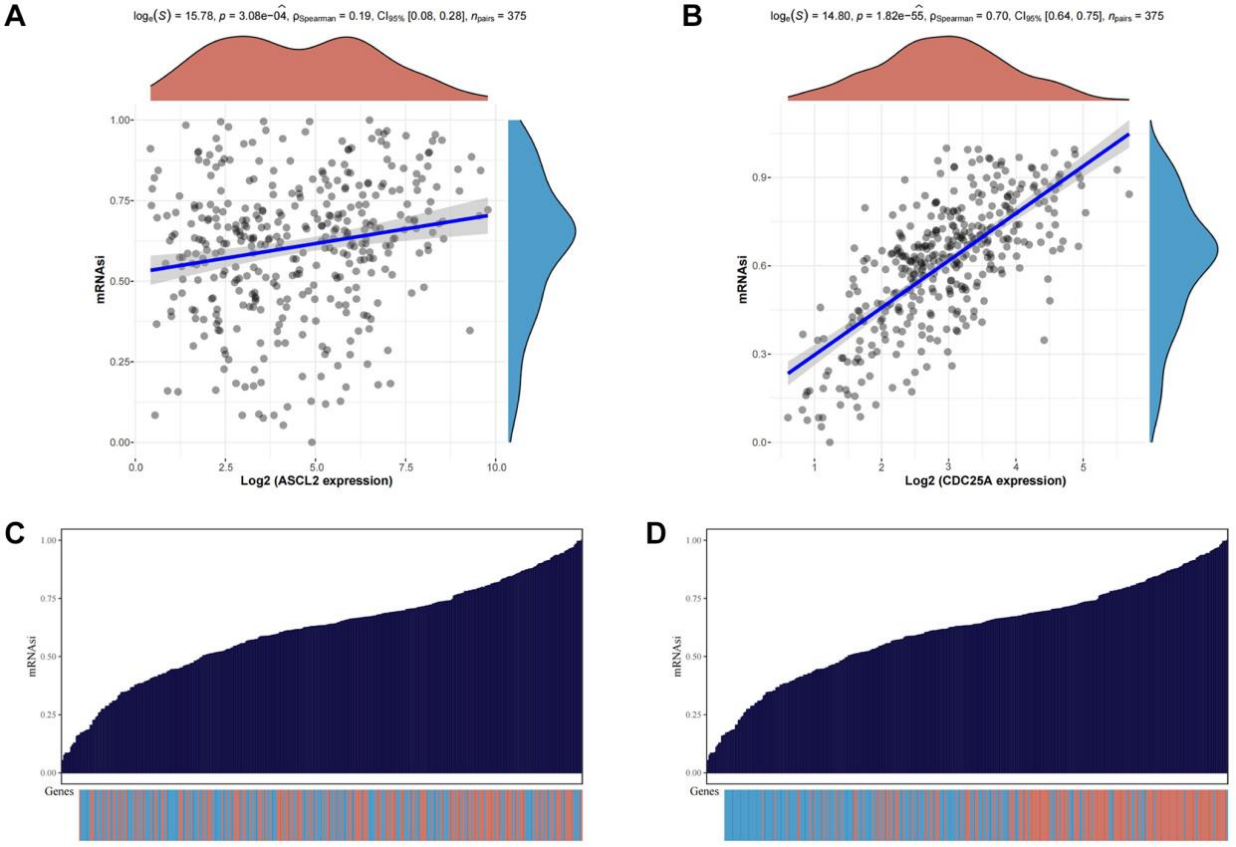
**Analysis of the relationship between TSRGs and tumor stemness.** (**A**) Scatter plot displaying the correlation between ASCL2 and the tumour stemness index. (**B**) Scatter plot displaying the correlation between CDC25A and the tumour stemness index. (**C**) Heat map of ASCL2 expression distribution versus tumor stemness score of corresponding samples. (**D**) Heat map of CDC25A expression distribution versus tumor stemness score of corresponding samples.

To probe the functional role of TSRGs, we conducted an analysis examining their expression and correlation with mRNAsi. The scatter plot unveiled a notably stronger correlation between CDC25A and the tumor stemness index compared to ASCL2 (Figure 10A, 10B). Moreover, the distribution patterns of stemness scores and gene expression underscored CDC25A’s closer association with escalating levels of mRNAsi compared to ASCL2 (Figure 10C, 10D). Consequently, CDC25A emerged as the prime candidate for further investigation.

**Figure 11:**
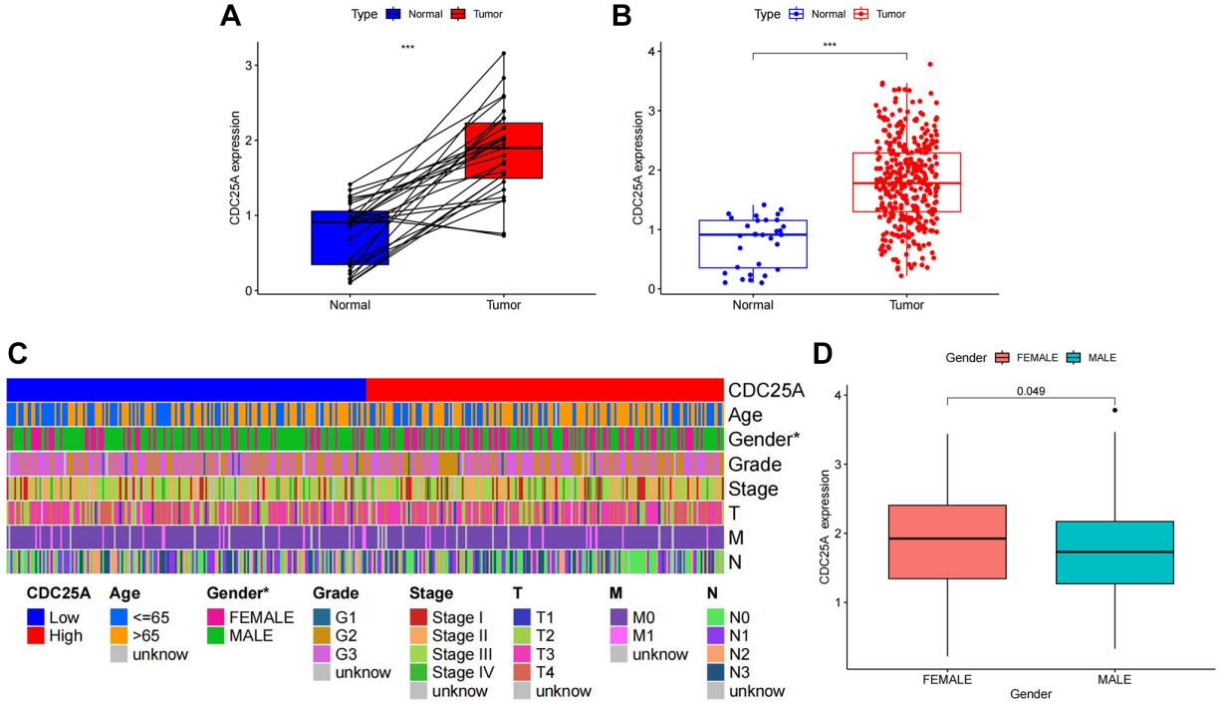
**Analysis of CDC25A expression in gastric cancer.** (**A**) Paired expression analysis of CDC25A in gastric cancer. (**B**) Analysis of CDC25A expression in gastric cancer. (**C**) Clinical heatmap of CDC25A expression in gastric cancer. (**D**) Differences in CDC25A expression between genders.

**Figure 12:**
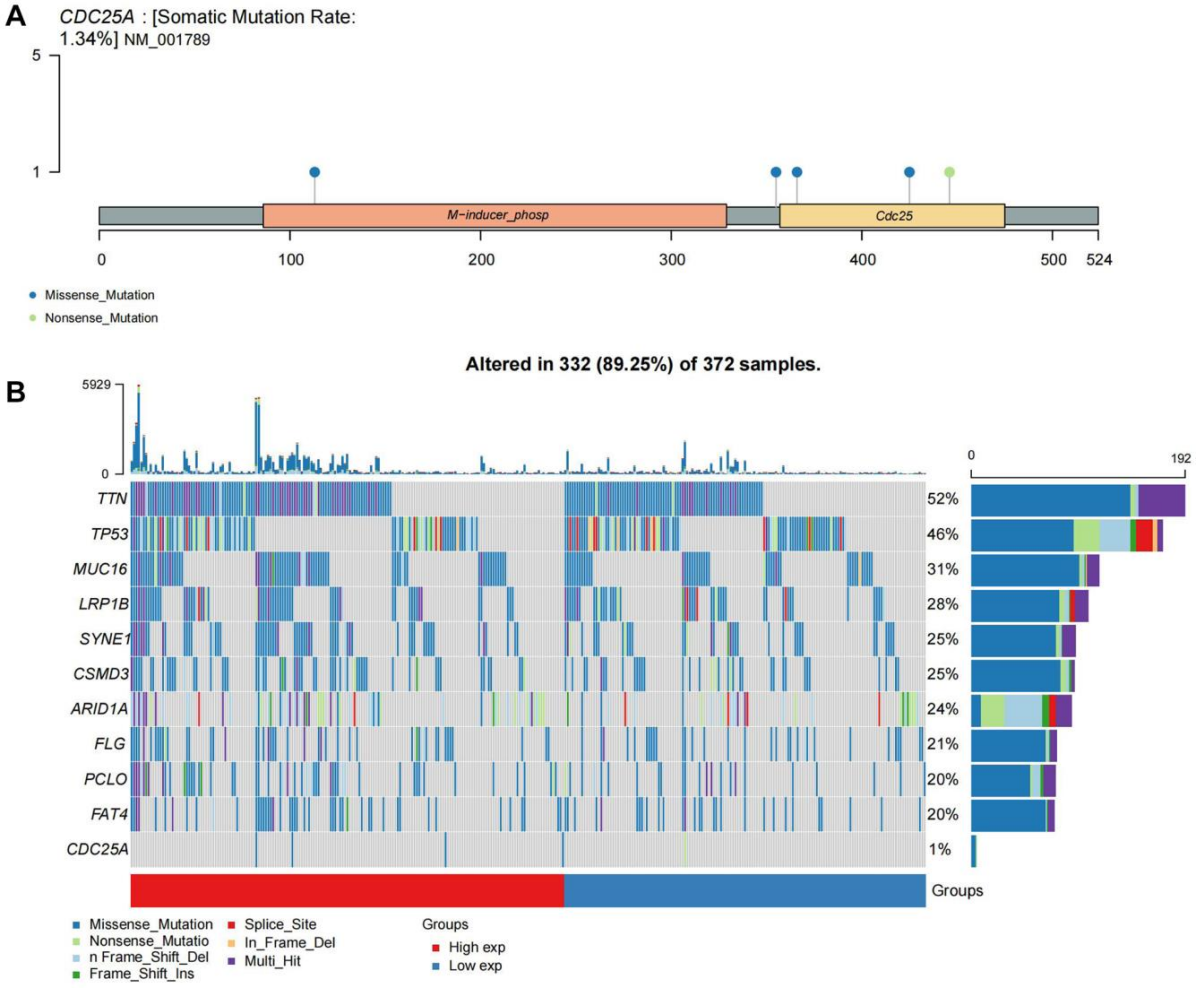
**Mutational landscape of CDC25A.** (**A**) Mutation sites and types of CDC25A gene. (**B**) Mutational landscape of CDC25A high and low expression groups in gastric cancer.

CDC25A expression exhibited a noteworthy upregulation in tumor tissue samples, as depicted in Figure 11A, 11B. Additionally, clinical heatmap analysis indicated a significant gender-based disparity in CDC25A expression, as illustrated in Figure 11C. Subsequent analysis revealed a marked elevation in CDC25A expression among female patients, as depicted in Figure 11D. Given the presence of mutations in multiple genes within cancer cells, somatic mutations in CDC25A were investigated, revealing the presence of five mutations - one nonsense and four missense - in gastric cancer cells, as shown in Figure 12A. Further evaluation of CDC25A’s mutation status within the high and low expression groups uncovered a high prevalence of mutations in TP53 and other oncogenes among the high expression group of CDC25A, as illustrated in Figure 12B. This finding suggests a potential association between the malignant phenotype of the tumor and the heightened expression of CDC25A.

**Figure 13:**
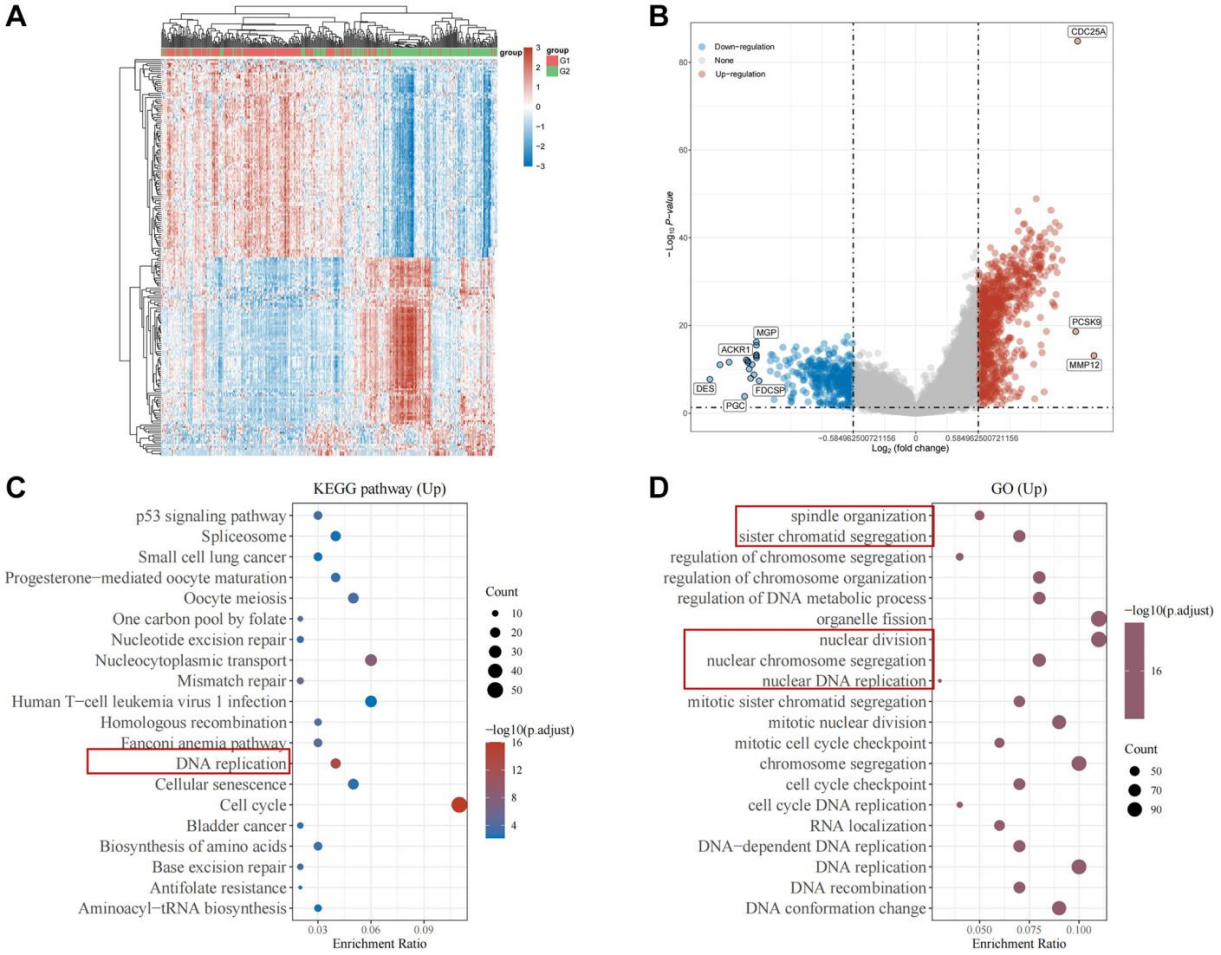
**Enrichment analysis of CDC25A in gastric cancer.** (**A**) Heat map of differential genes in the CDC25A high and low expression groups. (**B**) Differential gene volcano map of CDC25A high and low expression groups. (**C**) KEGG enrichment analysis of CDC25A. (**D**) GO enrichment analysis of CDC25A.

To elucidate the specific biological role of CDC25A within gastric cancer pathways, we conducted an enrichment analysis. Initially, we utilized the median expression value of CDC25A to identify differential genes in gastric cancer samples, as depicted in Figure 13A, 13B. Subsequent pathway enrichment analysis unveiled CDC25A’s involvement in the regulation of cell cycle and cell division in gastric cancer, as shown in Figure 13C. Functional enrichment analysis further underscored CDC25A’s regulatory role in critical cellular processes such as mitosis, sister chromatid segregation, spindle assembly, and DNA replication, indicative of the self-renewal and replication abilities of tumor stem cells, as depicted in Figure 13D.

**Figure 14:**
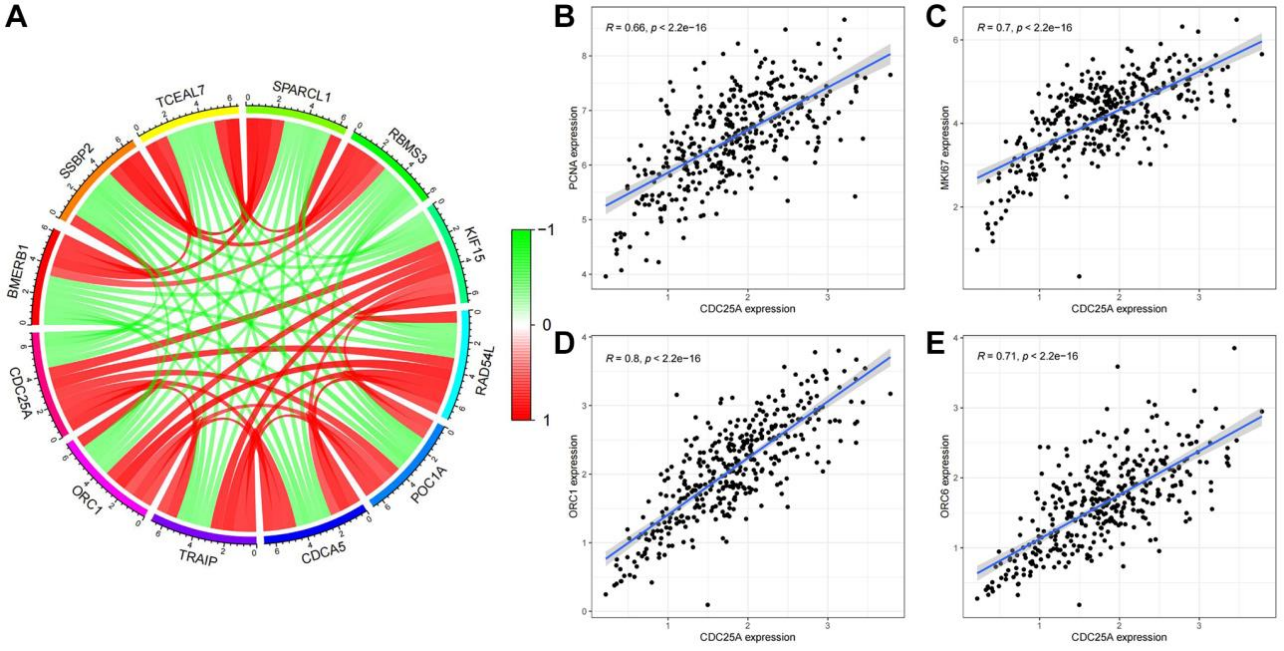
**Co-expression analysis of CDC25A in gastric cancer.** (**A**) Co-expressed genes of CDC25A. (**B**) Co-expression relationship between PCNA and CDC25A. (**C**) Co-expression relationship between MKI67 and CDC25A. (**D**) Co-expression relationship between ORC1 and CDC25A. (**E**) Co-expression relationship between ORC6 and CDC25A.

To validate these findings, we assessed the co-expression of genes with CDC25A CDC25A (Figure 14A). Notably, cell proliferation markers PCNA and MKI67 exhibited significant correlations with CDC25A, as depicted in Figure 14B, 14C. Furthermore, proteins OCR1 and ORC6, pivotal for DNA replication, demonstrated a high correlation with CDC25A expression, as shown in Figure 14D, 14E. Lastly, we conducted a correlation analysis to explore the relationship between CDC25A expression and signaling pathways associated with the cell cycle and proliferation. The results indicated a strong and positive association between CDC25A expression and DNA replication, tumor proliferation, G2M checkpoint, and MYC signaling pathways, as depicted in Supplementary Figure 6A–6D.

### CDC25A possesses the capability to regulate the proliferation of cells and the stemness of tumours

**Figure 15:**
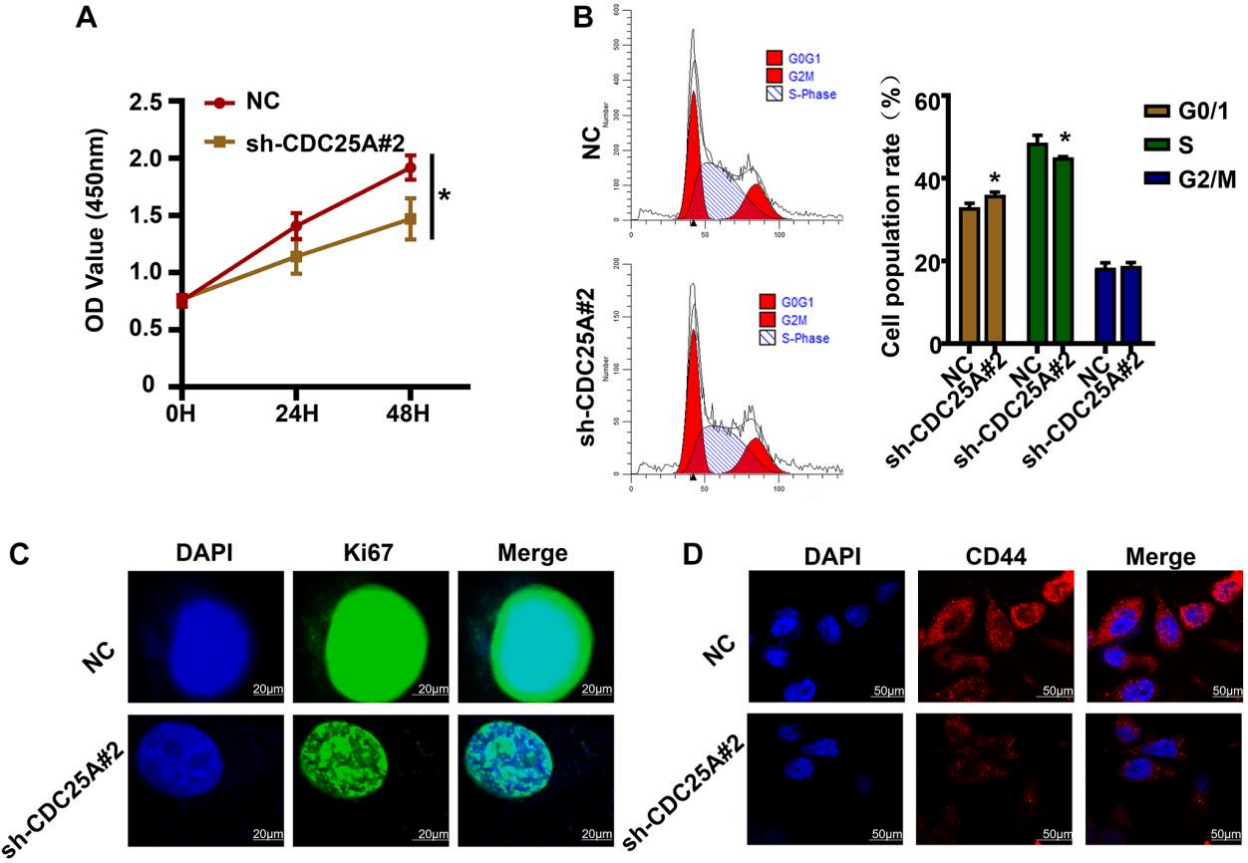
**The effects of CDC25A expression on the proliferation, cell cycle and tumor stemness of gastric cancer cells were verified *in vitro*.** (**A**) CCK8 assay to detect changes in gastric cancer cell viability after knockdown of CDC25A. (**B**) Flow cell cycle assay was performed to detect the changes in the percentage of cells in each cycle of gastric cancer cells after knockdown of CDC25A. (**C**) Cellular immunofluorescence assay to detect changes in MKI67 expression after CDC25A knockdown. (**D**) Cellular immunofluorescence assay to detect changes in CD44 expression after CDC25A knockdown.

In light of the bioinformatic analyses conducted, *in vitro* studies were carried out to verify the effects of CDC25A on gastric cancer cell proliferation, cell cycle, and tumor stemness. The CCK8 experimental findings indicated that suppressing CDC25A expression led to a noteworthy decline in the viability of gastric cancer cells by 0.4467 ± 0.1209 at 48 h (Figure 15A). The cell cycle results indicated that inhibiting CDC25A expression led to an increase in the proportion of cells in the G0/1 phase and a decrease in the number of cells in the S phase, while the proportion of cells in the G2 phase remained unchanged (Figure 15B). Co-expression analysis unveiled a significant positive correlation between CDC25A and MKI67. This finding was substantiated by our immunofluorescence results, demonstrating a marked decrease in MKI67 expression subsequent to CDC25A inhibition. (Figure 15C). To explore the impact of CDC25A on tumor stemness within gastric cancer, we selected CD44 as a well-established marker for cancer stemness investigation. As illustrated in Figure 15D, inhibition of CDC25A expression led to a notable decrease in CD44 expression. These experimental findings provide compelling evidence that CDC25A possesses the capacity to modulate both cell proliferation and tumor stemness in the gastric cancer.

## DISCUSSION

Patients diagnosed with gastric cancer have a bleak prognosis, while the underlying pathomechanisms of the disease are yet to be fully understood. Recent investigations underscore the pivotal role of tumor stem cells in confounding therapeutic endeavors aimed at combating the disease. Leveraging the power of high-throughput sequencing and microarray technology, we embarked on a comprehensive exploration of tumor stemness key genes in gastric cancer, employing advanced bioinformatics and machine learning methodologies. Subsequent meticulous analysis and validation of these genes *in vitro* shed light on their biological functions within the gastric cancer milieu. By elucidating the precise connection between tumor stemness and prognosis in gastric cancer, this study offers invaluable insights poised to inform therapeutic strategies and prognostic assessments in clinical practice.

In this study, we initially screened gastric cancer tumor stemness genes using ssGSEA and WGCNA jointly. Next, we screened TSKGs by applying various machine learning algorithms. These machine learning results were reviewed for accuracy and reliability. Finally, we selected the results of SVM screening to identify tumor stemness risk genes and construct a TSRGPM.

ASCL2 is a member of a family of transcription factors that play a role in several aspects of tumours. Most reports on its regulation of tumour stemness have appeared in colon cancer and in gliomas. For example, Wang, Li-Hong et al. reported that ASCL2 can control the tumour stemness phenotype of glioma cells by regulating the expression of ATG9 protein in glioma cells [19]. It has also been reported that ASCL2 is highly expressed in mismatch repair-sufficient or microsatellite-stabilised colon cancers and can maintain the stemness of colon cancer tumour cells and activate cancer-associated fibroblasts through the activity of transcription factors, thereby inducing an immune-rejecting microenvironment to inhibit the infiltration of immune cells [20]. CDC25A belongs to the CDC25 phosphatase family and is required for progression from the G1 phase to the S phase. Our experimental results consistently demonstrate that inhibiting the expression of CDC25A results in a decrease in the percentage of cells in the S phase during cell cycle experiments. Additionally, CDC25A contributes to the development of pancreatic cancer, colon cancer, hepatocellular carcinoma, and other cancers [21–24].

The TSRS derived from our constructed model exhibited a remarkable ability to discern between patients’ risk profiles and survival outcomes. Notably, a pronounced discrepancy in overall survival (OS) emerged between the high and low TSRS groups, with the latter indicative of potentially extended OS durations. Encouragingly, both Cox regression and ROC analyses substantiated the robust prognostic utility of TSRS. Through a comprehensive assessment integrating TSRS with various clinicopathological characteristics, we uncovered a notable disparity in TSRS grading concerning lymph node metastatic parameters (N). Consequently, a meticulous statistical scrutiny of TSRS distribution across distinct N grades was undertaken. Intriguingly, our findings unveiled a predominance of patients with high TSRS scores within the N1 grading, juxtaposed with a larger contingent of patients exhibiting low TSRS scores in the N0 category. Given the pivotal role of tumor stemness in driving cancer metastasis, our clinical heatmap analysis strikingly underscores the association between elevated TSRS scores and heightened incidence of lymph node metastasis in gastric cancer, affirming the clinical relevance of our findings. The precision and reliability of TSRS in delineating patient risk profiles were further underscored by these compelling observations.

In addition to the structural support of the tumour microenvironment for tumour development, the TME may also create favourable conditions for the formation and maintenance of the CSCs niche and regulate the self-renewal and other stem cell-like properties of CSCs. Therefore, we also analysed the relationship between TSRS and immune infiltrating cells. It is noteworthy that, taken together, several different algorithms show that the tumour immune infiltrating cells positively associated with TSRS include M2-type macrophages. Tumor-associated macrophages account for about 50% of the tumours in TME and predominantly exhibit an M2 phenotype, regulating tumour growth, migration and angiogenesis by producing a plethora of growth factors, cytokines and ECM remodelling molecules (e.g., CCL2, CXCL12, CXCR4, TGF-β, VEGF, PDGF, COX-2, and metalloproteinases), which contribute to the progression of almost all tumours [25]. In various cancer types, CSCs can promote macrophage recruitment through different molecular mechanisms [26]. For example, in glioblastoma and cholangiocarcinoma, CSCs can also recruit macrophages by specifically secreting POSTN that binds to the macrophage surface receptor integrin αvβ3 [27, 28]. CSCs not only promote macrophage recruitment, but also influence macrophage polarisation status. CSCs preferentially express and secrete the Wnt-induced signaling protein 1 (WISP1) and maintain M2 macrophage survival by activating the integrin α6β1/AKT pathway on macrophages via the paracrine pathway. Similarly, Liao et al. [29] further demonstrated that WISP1 was associated with the polarisation and maintenance of M2-type TAMs in various types of tumours, revealing that the regulation of M2-type TAMs by WISP1 in different tumours may be a universal phenomenon. Similarly, in ovarian cancer [30], bladder cancer [31], glioblastoma [32] and breast cancer [33], IL-6 and IL-10 secreted by CSCs converted TAMs to M2 phenotype. In addition to secreting factors, CSCs also regulate macrophage polarisation through exosome release. Like normal stem cells, CSCs exist in a cellular ecotope composed of multiple cell types, such as immune cells, MSCs, endothelial cells, and CAFs, which collectively provide a unique microenvironment that protects and promotes the function of CSCs through the secretion of a variety of cytokines to promote tumourigenesis, angiogenesis, invasion, metastasis, and drug resistance [34]. TAMs, as CSCs important immune cells in the ecological niche, can regulate the maintenance of CSCs and their ecological niche through the following signalling pathways and cytokines. IL-6 regulates tumour proliferation and differentiation mainly by mediating the IL-6/STAT3 signaling pathway. IL-6 secreted by TAMs promotes the transformation of non-CSCs to CSCs and the self-renewal of CSCs through the activation of STAT3. TAMs can promote tumour migration, invasion, and malignant progression by paracrine TGF-β. Ye et al. [35] found that macrophages enhanced the invasion of glioma stem cell-like cells through the TGF-β1 signalling pathway. In addition, epithelial-mesenchymal transition (EMT) is an important process that enables cancer cells to acquire CSCs-like characteristics and maintain CSCs stemness. M2-type TAMs induce EMT in hepatocellular carcinoma cells through secretion of TGF-β1, which results in higher invasive capacity and enhanced CSCs characteristics.

In the final phase of our investigation, CDC25A emerged as a focal point for in-depth exploration through *in vitro* experimentation, aimed at corroborating its impact on gastric cancer cells and tumor stemness. Remarkably, our findings revealed that the suppression of CDC25A expression in gastric cancer cells elicited a notable reduction in cellular viability and proliferation. Additionally, inhibition of CDC25A expression precipitated a discernible alteration in cell cycle dynamics, characterized by a decrease in the proportion of cells traversing the S-phase coupled with a concomitant increase in cells arrested at the G0/1 phase. These observations underscore the pivotal role of CDC25A in modulating the malignant behavior of gastric cancer cells, thereby offering promising avenues for targeted therapeutic interventions.

While our study successfully validated the efficacy of the established risk-prognostic model, certain limitations warrant acknowledgment. Specifically, the validation of patient distribution in the high TSRS group within the N1 classification remains unverified due to constraints in obtaining clinical samples. Moreover, a comprehensive array of molecular and animal experiments is imperative to elucidate the intricate molecular mechanisms governing the role of TSRGs in tumor stemness regulation. This imperative shall guide the central focus and trajectory of our future research endeavors.

In summary, our investigation has yielded a robust TSRGPM, wherein TSRS emerges as a potent prognostic indicator effectively stratifying patients’ risk profile. The elucidation of this risk model alongside the identification of associated risk genes furnishes a solid theoretical foundation for the implementation of precise individualized treatment strategies and the pursuit of novel therapeutic targets, thereby fostering advancements in the clinical management of gastric cancer.

## Supplementary Materials

Supplementary Figures
