# Supplementary material for: Machine learning for identifying tumor stemness genes and developing prognostic model in gastric cancer
Source: Aging (Albany NY). 2024 Apr 12;16(7):6455–77. doi: 10.18632/aging.205715 (PMC11042969; doi:10.18632/aging.205715)
Supplement: Supplementary Figures [file aging-16-205715-s001.pdf]

SUPPLEMENTARY FIGURES

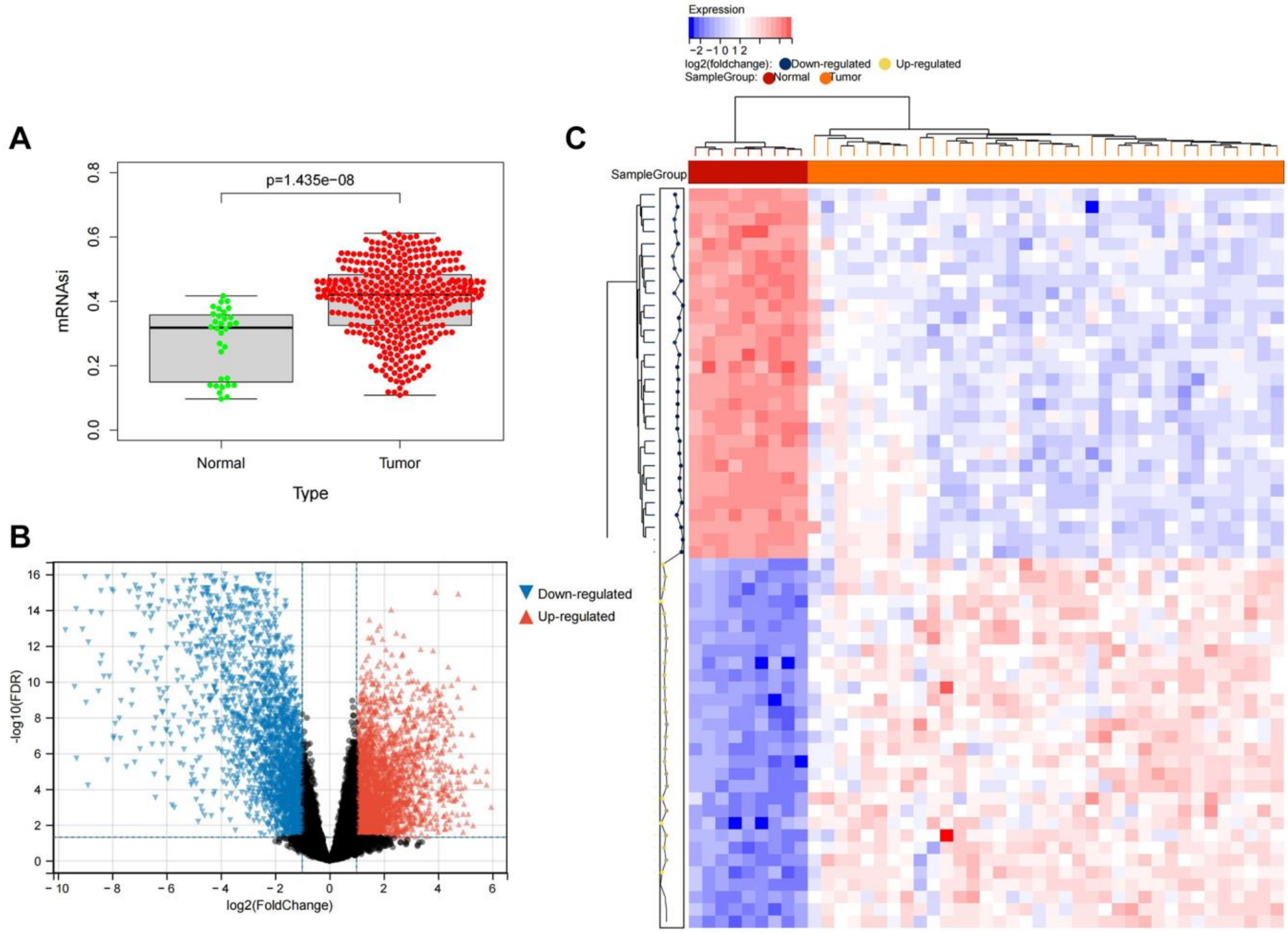

Supplementary Figure 1. Differences in mRNAsi and gene expression. (**A**) Differences in mRNAsi between normal and tumor tissues in gastric cancer. (**B**) Volcano map of differentially expressed genes. (**C**) The top 50 differentially expressed genes in gastric cancer disease presented as a gene expression heat map. $P < 0.05$.

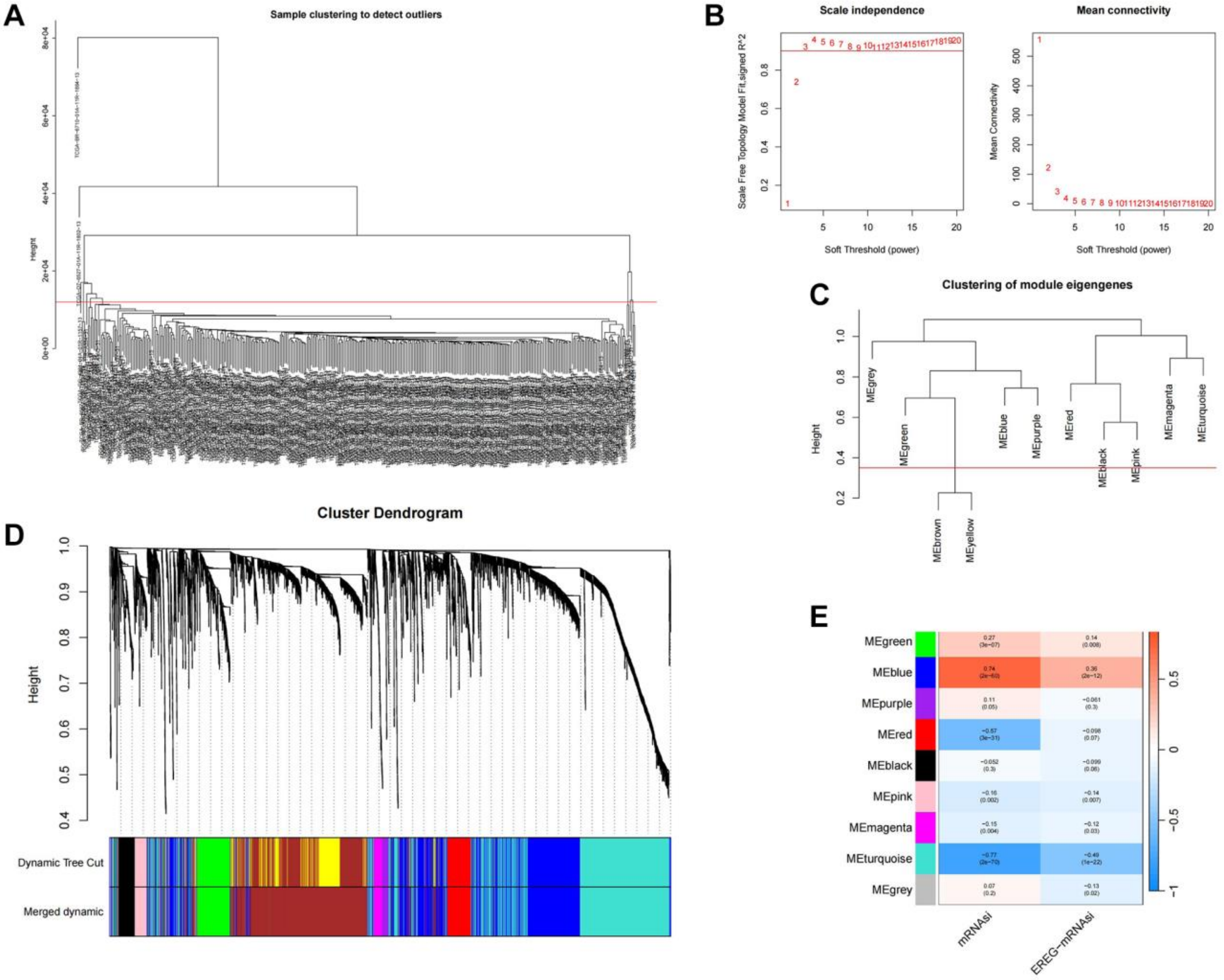

Supplementary Figure 2. Identification of cancer stemness index-related modules by WGCNA. (**A**) Samples above the red line were removed because they were considered as the deflection of gene expression. (**B**) This represents the correlation coefficient R^2 and mean connectivity in the scale-free network. (**C**) Calculate similarity between modules and merge modules with high similarity. (**D**) Hierarchical clustering of gene modules. (**E**) Heatmap of the correlation ship between gene modules and cancer stemness index.

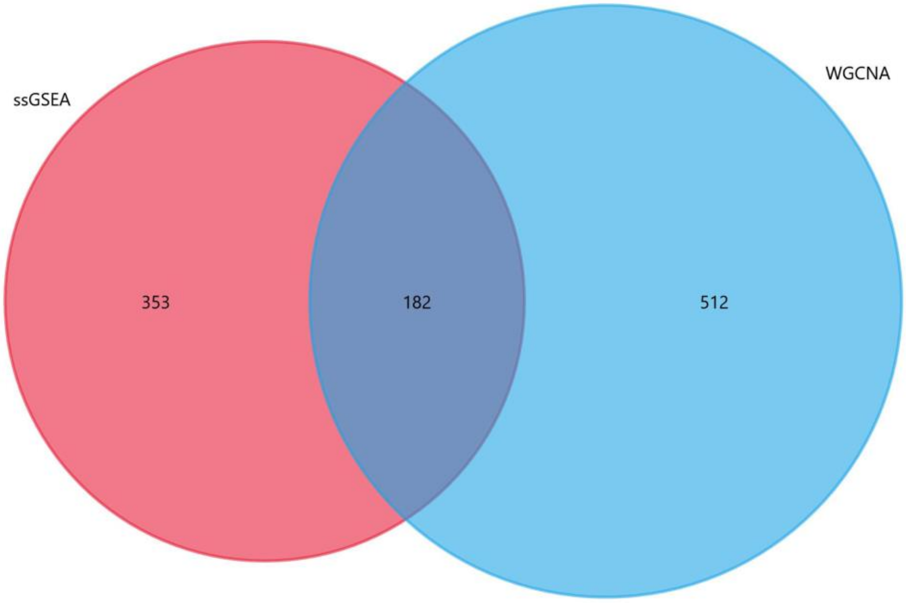

Supplementary Figure 3. Venn plot of genes from WGCNA versus genes from ssGSEA.

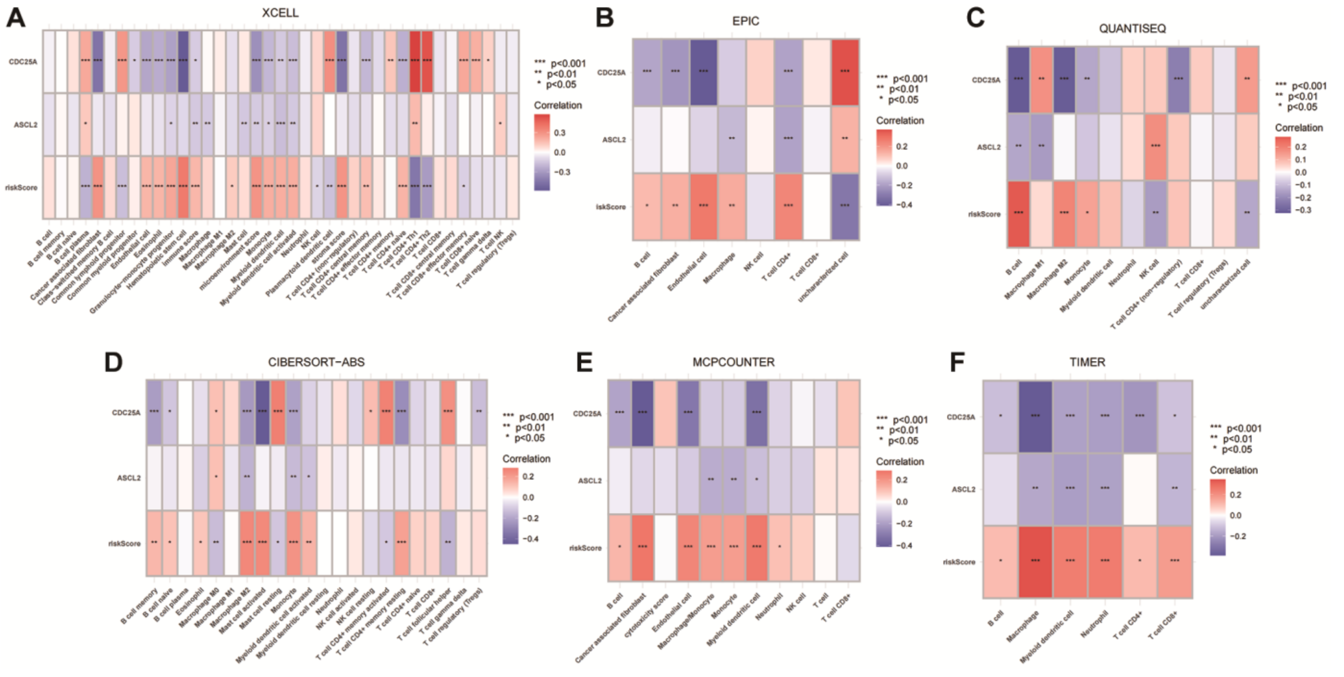

Supplementary Figure 4. Different algorithms for immune infiltration analysis show the relationship between TSRS and immune cells. (**A**) Result of XCELL algorithm; (**B**) MCPCOUNTER algorithm results; (**C**) Result of EPIC algorithm; (**D**) Result of CIBERSORT-ABS algorithm; (**E**) Result of QUANTISEQ algorithm; (**F**) Result of TIMER algorithm.

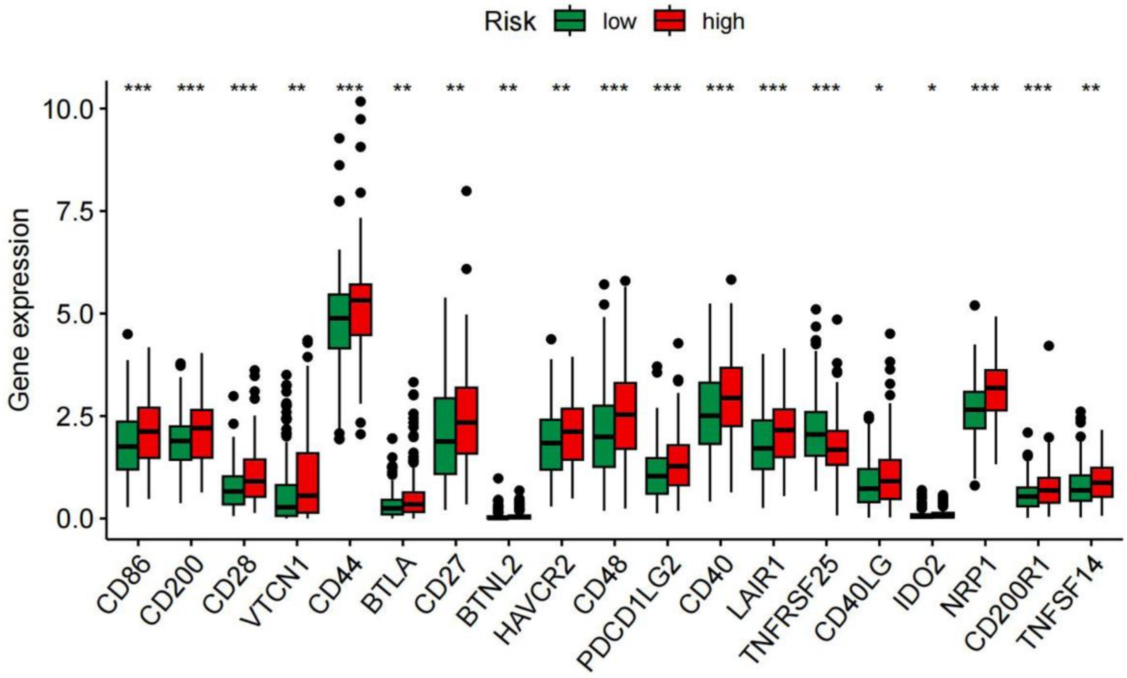

Supplementary Figure 5. The expression of immune checkpoint genes in high and low TSRS groups.

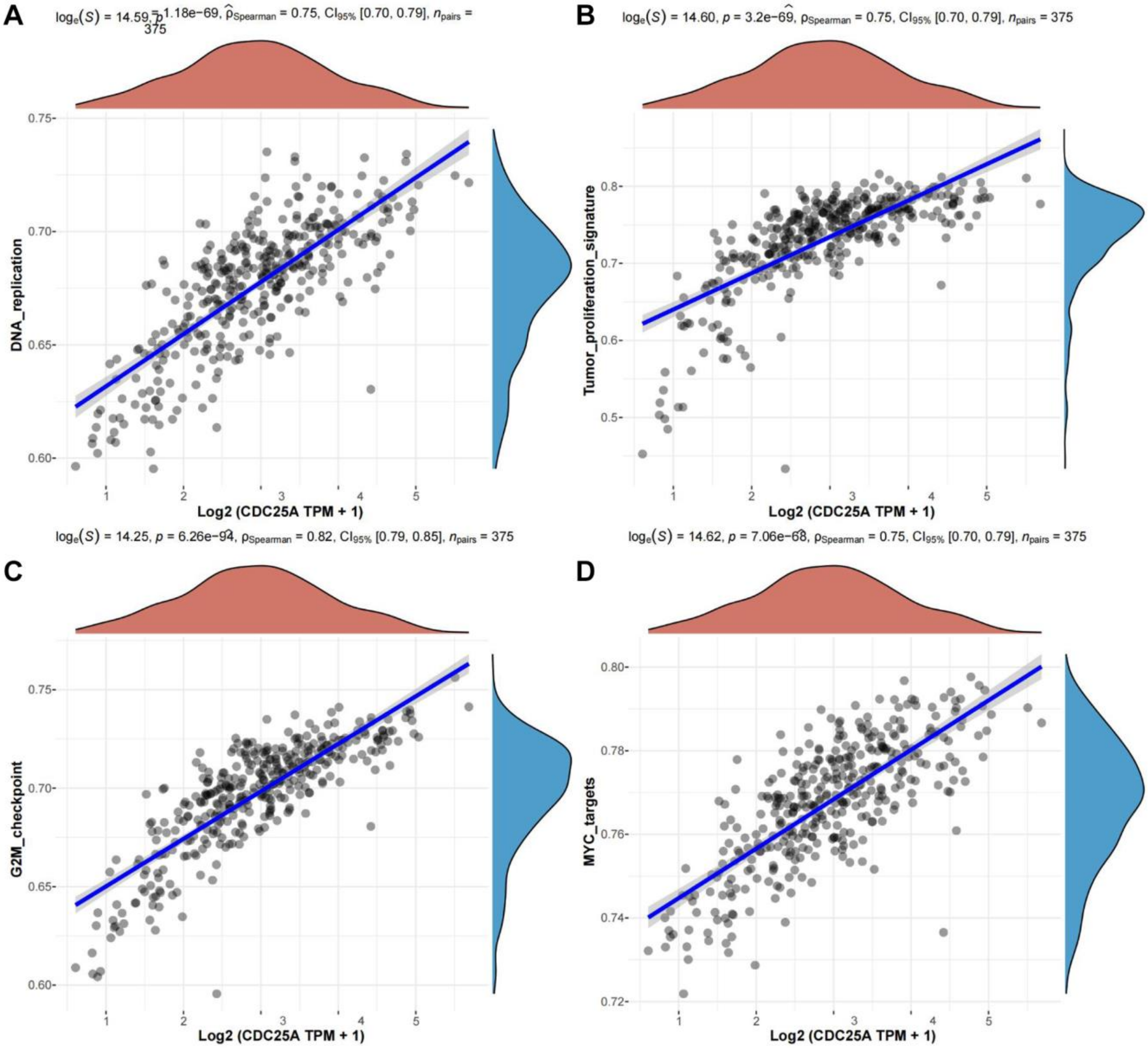

Supplementary Figure 6. Relationship between CDC25A expression and different signalling pathways. (**A**) Relationship between CDC25A expression and DNA replication signalling pathway. (**B**) Relationship between CDC25A expression and tumour proliferation signalling pathway. (**C**) Relationship between CDC25A expression and the G2/M cycle checkpoint signalling pathway. (**D**) Relationship between CDC25A expression and the MYC signalling pathway.
